# The Room Temperature Fracture Behaviors of GNPs/TA15 Composites by Pre-Sintering and Hot Extrusion

**DOI:** 10.3390/ma16010318

**Published:** 2022-12-29

**Authors:** Jiabin Hou, Wencong Zhang, Guorong Cui, Wenzhen Chen, Xing Wang, Shuo Wu, Qiang Ma

**Affiliations:** 1 Marine College, ShanDong JiaoTong University, Weihai 264209, China; 2 School of Materials Science and Engineering, Harbin Institute of Technology at Weihai, Weihai 264209, China; 3 China Aviation Industry Standard Parts MFG Co., Ltd., Guiyang 550014, China

**Keywords:** GNPs/TA15 composites, dislocation, microcracks, tensile test

## Abstract

Graphene nanoplates (GNPs)/TA15 composites were fabricated by pre-sintering and hot extrusion. During a room temperature tensile test, the dislocation was generated in grains. With increasing strain, the dislocation piled up along the interface between GNPs and Ti matrix, leading to stress concentration and microcracks. Then, the microcracks extended to GNPs or along the interface. The GNPs cracked under the shear force and the GNPs pulled out along with the crack propagation along the interface. This work provides a new sight in the room temperature tensile fracture behaviors.

## 1. Introduction

Graphene nanoplatelets (GNPs) have significant advantages on mechanical properties and specific surface area [1,2,3] and were identified as one of best reinforcements for metals [2,4]. Generally, GNPs had been widely used to reinforce Al [5,6,7], Mg [8,9], Cu [10,11,12], and Ti [13,14,15], obtaining excellent properties such as ultimate strength, ductility, abrasive resistance, etc. It is worth noting that strengthening was mainly achieved by the load transfer from matrix to the reinforcement components. In addition, the fracture behaviors gave a deep insight into understanding the load transfer process and provided a reasonable basis for the interface between GNPs and Ti matrix. Mu et al. [16] fabricated Ni-GNFs/Ti composites by spark plasma sintering (SPS) and hot-rolling. An in-situ tensile testing was induced to observe the failure mode. During the tensile test, micro-voids initiated around the cracked Ni-GNFs, and then extended to long cracks. However, the approach of in-situ tensile testing could only observe the designated area. It is hard to state the overall fracture process accurately. In addition, there was no characterization analysis for microcracks initiation. Moreover, the TEM measurement was also used to observe the microstructure and morphology of composites with 5% strain, i.e., the dislocation density along the interface increased significantly, and the microcracks to the interface between GNPs and Ti matrix appeared [17]. They considered that the dislocation accumulation induced local stress concentration, resulting in microcracks initiation. Here, the microcracks initiation were studied, while the fracture process and cracks propagation were not investigated further. In our previous work [18], a simple method had been successfully used for qualitatively analyzing the fracture process of TiBw/TA15 composites with SEM. Meanwhile, electron back scattered diffraction (EBSD) measurements could be carried out to characterize microstructure evolution, i.e., the kernel average misorientation (KAM) results could display the dislocation multiplication and motion.

Herein, the GNPs/TA15 composites were pre-sintered at 900 °C and hot extruded at 900 °C. A new method was introduced to investigate the room temperature fracture behaviors, combining SEM and EBSD. It is beneficial to reveal the fracture behaviors for optimizing the properties of GNPs/Ti composites. 

## 2. Materials and Methods

The GNPs/TA15 composites were fabricated by pre-dispersing, pre-sintering, and hot extrusion. The details are as follows: (I) Pre-dispersing: TA15 is a type of α titanium alloy with the advantages of mechanical properties and heat resistance, and its composition are shown in Table 1. The 0.4 wt.% GNPs (D50 ≈ 6.423 µm, thickness ≤ 6 nm) and TA15 powders (Purity ≥ 99%, 120 µm) were low-energy milled, and absolute alcohol was added. The mixed powders were mechanically stirred, vacuum filtered, and vacuum dried to remove the liquid. After pre-dispersing, the GNPs distributed on the surface of TA15 powders. (II) Pre-sintering: The dried powders were cold-pressed and weld-sealed in a 45# steel can. A high-temperature furnace was employed to sinter the cans at 900 °C for 45 min, namely, pre-sintering. During pre-sintering, the interface reaction between GNPs and TA15 matrix occurred, and the necks among TA15 powders were formed, obtaining a net structure. (III) Hot extrusion: Once more, the air-cooled billets were heated in the furnace at 900 °C for 30 min. Then, the hot billets were extruded in 5 s, obtaining φ16 mm × 500 mm bars under a 10.6 extrusion ratio. After extrusion, the GNPs distributed along extrusion direction (ED), which was beneficial for bearing load during the tensile test.

Scanning electron microscopy (SEM, Zeiss-MERLIN) was employed to observe the microstructure and side-fracture along ED, which was fitted with an EBSD system. The interface and phases were analyzed by transmission electron microscopy (TEM, Talos F200S G2). Furthermore, the room temperature tensile tests were carried out at 10^−3^ S^−1^, using a universal testing machine (MTS) equipped with an extensometer.

## 3. Results and Discussion

**Figure 1:**
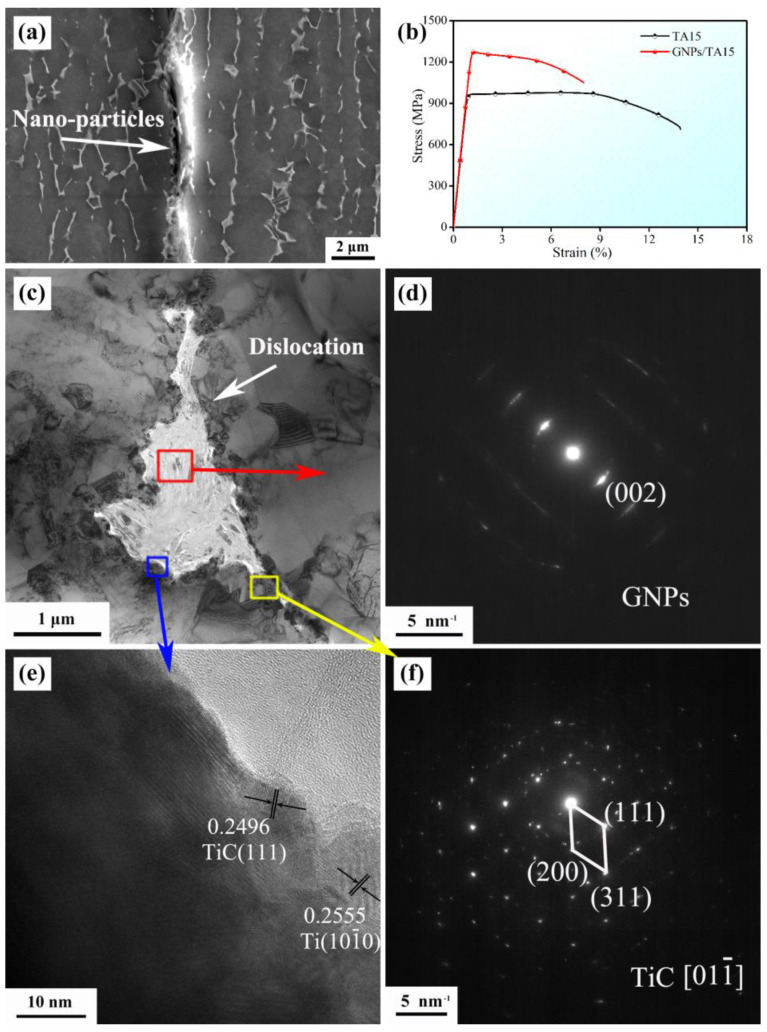
The SEM image along ED (**a**), room temperature stress-strain curve (**b**), TEM image along TD (**c**), selected area electron diffraction patterns (**d**,**f**), HR−TEM image (**e**).

The microstructure, phases, and mechanical properties of GNPs/TA15 composites are exhibited in Figure 1. Figure 1a presents elongated α grains, lamella phase along the extrusion direction (ED), along with nanoparticles distributed at the interface. It is worth noting that the interface structure and phases need to be identified, as shown in Figure 1c. The selected area electron diffraction (SAED) patterns were well-done in Figure 1d,f, i.e., (002) lattice plane of GNPs, and [01-1] crystal orientation of TiC. It is thus concluded that the bright phase highlighted by a red rectangle was made of GNPs, and the nanoparticles marked in a yellow rectangle were TiC. In addition, Figure 1e shows HR-TEM images of an area, highlighted by a blue rectangle. The (111) lattice plane of TiC and (10-10) lattice plane of α-Ti were standardized corresponding to interplanar crystal spacing. Moreover, nano-TiC particles formed between GNPs and Ti matrix, due to interface reaction, resulting in strong interface bonding.

With respect to GNPs/TA15 composites, the TA15 was similarly prepared by pre-sintering and hot extrusion at the same parameters. The room temperature stress–strain curves of GNPs/TA15 composites and TA15 are presented in Figure 1b. From the curves, the ultimate strength was significantly improved, increasing from 977 MPa to 1273MPa. This result shows that the addition of GNPs observably strengthened the TA15 matrix. Based on previous studies [15,19], three factors were mainly attributed to reinforcement, shown as follows: (I) Stress transfer: As we all know, GNPs have an obvious advantage on specific surface area and mechanical properties. It is thus considered that the large contact area formed between GNPs and Ti matrix results in a high stress transfer efficiency. In addition, GNPs have enough bear capacity due to excellent mechanical properties. Hence, it is no doubt that GNPs could bear the transferring stress from the Ti matrix efficiently. (II) Dislocation strengthening: GNPs and nano-TiC particles could effectively hinder dislocation motion. As a result, dislocation would pile up around them. Fortunately, this phenomenon was exhibited in Figure 1c, providing direct evidence for dislocation strengthening. (III) Solution strengthening: It is well-known that C atoms are one of the interstitials in Ti atoms, i.e., the solid solubility of C atoms was about 0.05 wt.% at room temperature, and the reinforcement was approximately 7 MPa/0.01 wt.%. Thus, the solution strengthening value was more than 35 MPa.

**Figure 2:**
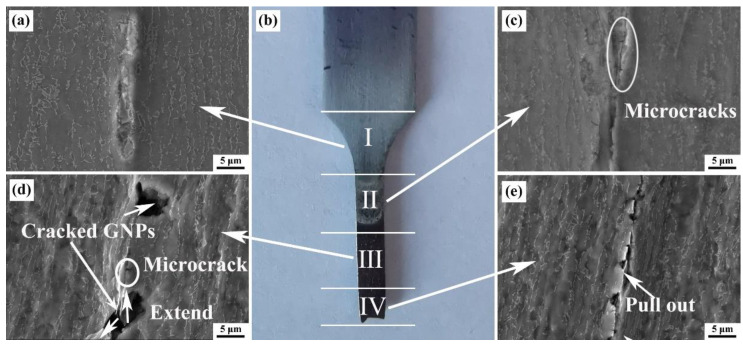
The side fracture specimen of GNPs/TA15 composites (**b**); SEM images with different locations (**a**,**c**–**e**).

The SEM images were employed to deeply investigate the fracture process of GNPs/TA15 composites, i.e., observing the side morphology of tensile specimens with different locations, as shown in Figure 2. Figure 2b presents the side areas of a fractured tensile specimen. It is considered that GNPs/TA15 composites belong to ductile fracture, concerning the necking region. To state the fracture process clearly, the side surface was divided into four sections [18]: I arc area. Figure 2a shows the yield stage and the system before the yield stage; II is close to the arc area, shown in Figure 2c, and is consistent with the minor plastic deformation stage; III is near fracture area, shown in Figure 2d, and corresponds to the major deformation stage; IV is the fracture area, where the fracture stage is shown in Figure 2e. Figure 2a shows the SEM image of area I, in which the microstructure was consistent with the one in Figure 1a. This indicates that during yield stage and before, GNPs and Ti matrix synchronously deformed along tensile direction. Nevertheless, microcracks distributed along interface are clearly observed in Figure 2c, during the minor plastic deformation stage. Moreover, the cracked and pulled-out GNPs are exhibited in Figure 2d. In addition, large cracks closed to the cracked GNPs, and microcracks appeared in the interface. Crack propagation could be responsible for this phenomenon. In the area near fracture, pulled-out and cracked GNPs were distinctly detected, and cracks distributed along GNPs. 

It is no doubt that the microcracks would form along the interface [10] and extend to GNPs, the Ti matrix, and the interface, when the deformation stepped into the plastic deformation stage. When the crack extended to the Ti matrix, the crack tip distinctly blunted wide, namely, the crack-tip-shielding mechanism occurred [20]. When the crack extended along the interface, the crack tip would also be hindered by nano-TiC particles, leading to pulled-out GNPs. Thus, the crack primarily extended to GNPs, i.e., the shear force would act on the GNPs. Based on the shear lag theory [21], the GNPs would crack until the shear force above the limiting stress of GNPs, resulting in cracked GNPs.

**Figure 3:**
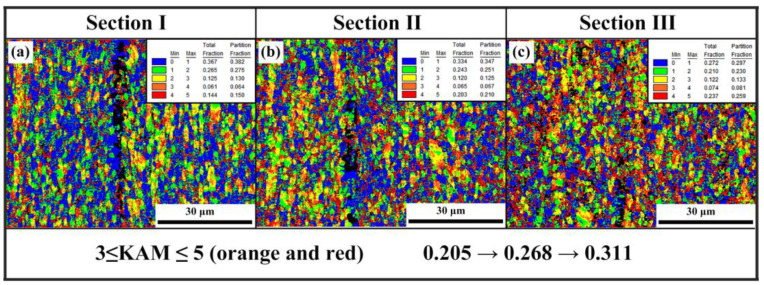
The KAM mappings of tensile specimen with different location: (**a**) I arc area, (**b**) II close to arc area, (**c**) III near fracture area.

The EBSD was adopted to analyze the microstructure evolution of GNPs/TA15 composites during a tensile test, i.e., KAM mappings with respect to different locations. The deformation of area IV was above the measured limiting of EBSD, resulting in an inaccurate value. Thus, area I, area II, and area III were deeply researched, as shown in Figure 3. It is observed that five kinds of colors represent different KAM values from zero to five, i.e., orange and red colors represented high dislocation density. From Figure 3a, the dislocation mainly distributed near grain boundaries. The color of the area adjacent GNPs (highlighted by black) was evidently blue, indicating the minimum dislocation density. This may be related to dynamic recrystallization (DRX) during hot extrusion. Figure 3b exhibits the KAM result of II area, i.e., the high dislocation density appeared near grain boundaries and GNPs, respectively. As shown in Figure 3c, the fraction of KAM value between three and five was up to 0.311 and concentrated on GNPs adjacent area. As the deformation increased, dislocation piled up along grain boundaries and the interface between the GNPs and the Ti matrix. Furthermore, the GNPs could effectively and efficiently hinder dislocation motion. Reasonably, the piled-up dislocation should be responsible for stress concentration, strengthening, and forming microcracks along the interface. 

**Figure 4:**
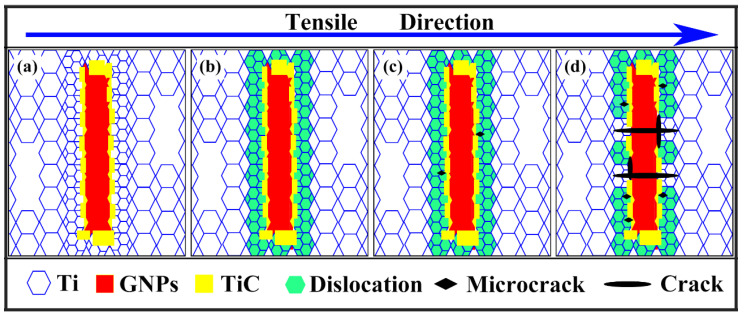
The schematic images of fracture process: (**a**) as-extruded composites, (**b**) yield stage, (**c**) minor plastic deformation stage, (**d**) major plastic deformation stage.

Figure 4 presents the schematic fracture process of GNPs/TA15 composites during the tensile test, i.e., the fracture behaviors were clearly stated. During deformation, a mass of dislocation piled up along the GNPs, due to their barrier effect [16,20]. The grains near GNPs would occur DRX [19], obtaining fine grains and annihilating the dislocation, as shown in Figure 4a. At the period of yield stage, the dislocation was generated in crystalline grains, particularly in the adjacent area of the GNPs. Then, the dislocation would move to GNPs and pile up gradually in Figure 4b due to inconsistent deformation between GNPs and Ti matrix [16]. Similarly, from Figure 4c, as the deformation stepped into minor plastic stage, the piled-up dislocation density increased rapidly, concentrating stress along the interface [17]. The initiation of microcracks was closely related to stress concentration. Finally, under tensile force, the microcracks extended to GNPs and were along the interface. When the cracks propagated along the interface, the in-situ TiC particle would impede them, resulting in pulled-out GNPs due to the strong bonding strength. It is thus reasonable that the GNPs would be subjected to the action of shear force [21] and the GNPs cracked along transverse direction, as shown in Figure 4d.

## 4. Conclusions

The room temperature fracture behaviors of GNPs/TA15 composites were researched by combining SEM and EBSD. During the tensile test, a mass of dislocation generated in grains and moved to GNPs, because the deformation between the Ti matrix and GNPs was not consistent with each other. The dislocation moved and piled up, leading to stress concentration and microcracks. With the increasing of strain, the microcracks mainly extended to GNPs and were along the interface, forming cracked and pulled-out GNPs. In addition, stress transfer, dislocation strengthening, and solution strengthening allowed the ultimate tensile strength to improve from 977 MPa to 1273 MPa with the addition of 0.4 wt.% GNPs.

## Figures and Tables

**Table 1:** The composition of TA15 powders.

Elements	Ti	Al	Mo	V	Zr
Content(wt.%)	89.997	6.24	1.00	1.61	1.96

## Data Availability

Data will be made available on request.
